# Identification of key genes associated with infertile endometriosis based on bioinformatic analysis

**DOI:** 10.3389/fgene.2025.1615268

**Published:** 2025-07-23

**Authors:** Linlin Chang, Hongjuan Ye, Min Hou, Xin Xie, Yang Wang, Jie Cheng, Rongxiang Wang, Xiaocong Chen, Xinxin Quan, Lihua Sun, Songguo Xue, Liya Shi

**Affiliations:** ^1^ Department of Obstetrics and Gynecology, Shanghai East Hospital, Tongji University School of Medicine, Shanghai, China; ^2^ The Shanghai Towako Hospital, Shanghai, China; ^3^ Department of Reproductive Medicine, International Peace Maternity and Child Health Hospital, Shanghai Jiao Tong University, Shanghai, China; ^4^ Department of Cardiology, Shanghai East Hospital, School of Medicine, Tongji University, Shanghai, China; ^5^ Department of Obstetrics and Gynecology, The First Affiliated Hospital of Anhui Medical University, Hefei, Anhui, China; ^6^ Center for Reproductive Medicine, Renji Hospital, School of Medicine, Shanghai Jiao Tong University, Shanghai, China; ^7^ Department of Reproductive Medicine Center, Shanghai East Hospital, Tongji University School of Medicine, Shanghai, China; ^8^ Department of Obstetrics and Gynecology, Ji ’an Hospital, Shanghai East Hospital, Tongji University School of Medicine, Shanghai, China

**Keywords:** endometriosis, infertility, differentially expressed genes, biomarkers, DEGs

## Abstract

**Background:**

Endometriosis is a common disease among women of childbearing age. However, the molecular mechanism behind it is still unknown. Therefore, new biomarkers and therapeutic targets are needed to improve the diagnosis and treatment of infertile women.

**Methods:**

Microarray datasets GSE7305, GSE7307, and GSE51981 were downloaded from the Gene Expression Omnibus database to identify differentially expressed genes (DEGs) between control and endometriosis. The STRING database and Cytoscape software constructed protein-protein interaction and hub gene networks. At the same time, the three data sets were screened for co-differentially expressed genes related to mitosis. Subsequently, we identified mitosis-related hub genes (MRHGs) associated with both mitosis-related genes and hub genes. Next, enrichment analysis for target genes was performed by Gene Ontology (GO) annotation and the Kyoto Encyclopedia of Genes and Genomes (KEGG) pathway, and the mRNA-miRNA network was constructed. Finally, GSE25628 and GSE6364 were used to verify the expression of MRHGs individually, while GSE120103 was employed to ascertain the influence of mitosis-related genes on female fertility.

**Results:**

A total of 93 DEGs were identified in the endometriosis datasets. Then, we placed 11 potential mitosis-related downregulated hub genes, among which eight showed good diagnostic properties of endometriosis, and two showed good diagnostic properties of infertile endometriosis. The main enriched GO functions revealed that the cell cycle mitotic pathway may be the critical pathway in endometriosis. Meanwhile, mRNA-miRNA interaction networks were constructed by choosing co-expressed mRNAs and miRNAs. Furthermore, cordycepin showed high drug-targeting relevance in infertile endometriosis.

**Conclusion:**

We identified eight mitosis-related hub genes as potential biomarkers for diagnosing and treating endometriosis. CENPE and CCNA2 might be associated with infertile endometriosis by affecting the endometrial secretory phase transition. In addition, cordycepin may be a potential clinical treatment for people with infertility-related endometriosis.

## Highlights

• This article uses bioinformatic methods to identify target genes for the treatment of endometriosis.
• This article adopts a new search method for target genes based on the common characteristics of infertility and endometriosis.
• Scan suitable therapeutic agents for target genes from the drug database.

## Introduction

The endometriosis prevalence seems to be considerably higher in sub-fertile women, ranging from 20% to 50%. The causes of infertility in women with endometriosis mainly involve anatomical distortions, endocrine abnormalities, and immune disorders (Taylor et al., 2021; Tanbo and Fedorcsak, 2017; Bonavina and Taylor, 2022). Implantation of the embryo depends on the endometrial inflammatory mechanism during pregnancy; endometriosis creates opportunities for chronic inflammation and disrupts endometrial receptivity, leading to infertility (Lessey and Kim, 2017).

Infertility is one of the most common problems among couples worldwide (Shaulov et al., 2020). The study found that implantation rate, clinical pregnancy rate, and ovarian response were lower in patients with endometriosis than in patients with tubal infertility (Zhong et al., 2021; Coccia et al., 2011; Al-Azemi et al., 2000; Boucret et al., 2020). Infertile patients with endometriosis impacted endometrial receptivity and endometrium decidualization (Kim et al., 2019). Human endometrium has a crucial role in the implantation process (Ranisavljevic et al., 2019). Endometrial deindividuation is regulated by the synergistic action of maternal steroid hormones, estrogen, and progesterone and is a necessary prerequisite for embryo implantation in early pregnancy. Uterine stromal cell mitosis plays a vital role in this process, and stromal cells must undergo mitotic expansion before full decidualization (Wang et al., 2010; Muter et al., 2015).

Endometriosis-associated infertility arises from the complex interplay of multiple mechanisms, including compromised oocyte quality, disrupted embryonic development, ovarian dysfunction, and diminished endometrial receptivity (Bonavina and Taylor, 2022). The WNT4/WNT5A genes, which serve as pivotal regulators in embryo implantation and uterine development, exhibit abnormal expression patterns that are closely linked to endometriosis. The WNT signaling pathway plays a critical role in the initiation and progression of endometriotic lesions by modulating cell proliferation, differentiation, and tissue remodeling processes (Gaetje et al., 2007; Sanchez et al., 2014). Current treatment strategies for endometriosis-related infertility have evolved beyond traditional lesion excision to encompass targeted therapies addressing the underlying pathogenic mechanisms. Recent studies emphasize the significance of regulating the hormonal milieu, attenuating inflammatory responses, and improving endometrial receptivity. Several novel pharmacological agents, such as letrozole, GnRH modulators, and progestogen-based drugs, demonstrate substantial therapeutic potential in clinical practice (Colombi et al., 2024). Furthermore, endometriosis exhibits a strong genetic predisposition and familial clustering tendency (McGrath et al., 2023).

Despite much evidence showing relationships between endometriosis and infertility, the mechanisms implicated in endometriosis-associated infertility are so far not fully understood. Transcriptomic methods ranging from large-scale RNA sequencing to single-cell/space technologies have become indispensable approaches in reproductive biology, including ovarian development, reproductive tract development, embryonic development, stem cell differentiation, and tissue regeneration (Gulati et al., 2025). They can help to analyze gene regulatory networks, accelerate the discovery of biomarkers for endometriosis, and reveal conserved pathways among plants, insects, and mammals (Li et al., 2024; Marečková et al., 2024). Therefore, screening differentially expressed genes between endometriosis and infertility may provide a new way to identify the pathogenesis of infertile endometriosis and give a new direction for treatment.

## Materials and methods

### Data collection

**FIGURE 1:**
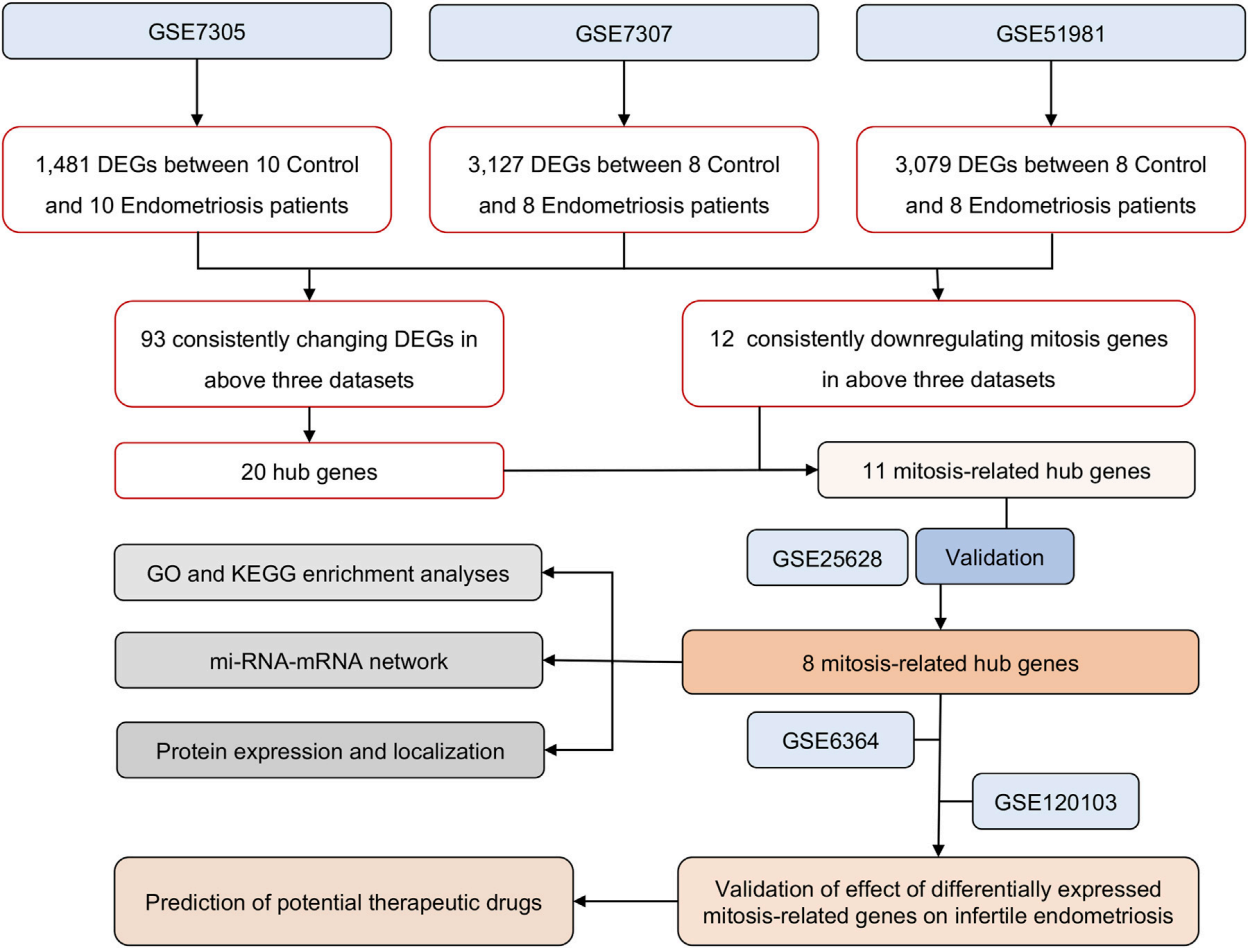
The overall protocol of this study. DEGs, differentially expressed genes; GO, Gene Ontology; KEGG, Kyoto Encyclopedia of Genes and Genomes; RIF, repeated implantation failure.

The NCBI GEO database (http://www.ncbi.nlm.nih.gov/geo/) offers a comprehensive and publicly accessible collection of functional genomic datasets and high-throughput microarrays based on next-generation sequencing. We downloaded gene expression data from this database for our study. (Clough and Barrett, 2016). We acquired six datasets related to endometriosis and endometriosis-related infertility from GEO. One of these datasets, GSE120103, pertained to endometriosis-related infertility, while the other five, namely, GSE7305, GSE7307, GSE51981, GSE25628, and GSE6364, were focused on endometriosis. The dataset named GSE7305 consisted of 20 samples, with 10 samples each of ectopic and normal endometrium. Another dataset, GSE7307, comprised eight normal endometrium samples and eight ectopic endometrium samples. Similarly, GSE51981 had eight normal endometrium samples and eight ectopic endometrium samples, while GSE25628 had six normal endometrium samples and six ectopic endometrium samples. In addition, GSE6364 had 11 normal endometrium samples and 10 ectopic endometrium samples. Finally, GSE120103 contained four normal endometrium samples from fertility and four ectopic endometrial samples from infertility. Following the transformation of identity documents, in cases where more than one probe matched a single gene, the gene expression value was determined by calculating the average expression value. Before analysis, raw data underwent log2-transformation and quantile-normalization. Table 1 provides detailed information on all six datasets, while the study design is presented as a flow chart in Figure 1.

**TABLE 1:** Gene expression datasets used in this study.

Accession	Platform	Experiment type	Control(n)	Endometriosis (n)	Infertile endometriosis (n)	Sample type
GSE7305	GPL570	mRNA array	10	10		Endometrium
GSE7307	GPL570	mRNA array	8	8		Endometrium
GSE51981	GPL570	mRNA array	8	8		Endometrium
GSE25628	GPL571	mRNA array	6	6		Endometrium
GSE6364	GPL570	mRNA array	11	10		Endometrium
GSE120103	GPL6480	mRNA array	4		4	Endometrium

### Identification of DEGs

The microarray data from four datasets was downloaded and analyzed using various statistical techniques. Principal component analysis (PCA) was performed to verify the data’s reproducibility. The differential expression analysis was carried out utilizing the 'limma’ package of R software (Davis and Meltzer), and genes with an adjusted P-value less than 0.05 and absolute fold change more significant than one were considered differentially expressed genes (DEGs). To visualize the results, various plots such as box line plots, heat maps, and volcano maps were generated using the 'ggplot2’ and 'heatmap' (version 3.3.6) packages of R software (version 4.2.1) (Gu et al., 2016).

### PPI network construction and module analysis

In this study, we used the Search Tool for the Retrieval of Interacting Genes (STRING) version 12.0 online database to predict the protein-protein interaction (PPI) network of differentially expressed genes (DEGs). The PPI network provides valuable insights into the generation or development of diseases. We considered interactions with a combined score greater than 0.4 statistically significant and used Cytoscape version 3.9.1, an open-source bioinformatics software platform, to visualize the network (Smoot et al., 2011). The Cytoscape software was utilized to import and analyze STRING data. The MCC algorithm of the Cytohubba plugin was employed to identify the top 20% scoring genes, which were then labeled as hub genes. Mitosis-related genes from the GeneCards database were used to match with the hub genes, resulting in the identification of overlapping genes associated with mitosis.

### Construction of an mRNA-miRNA regulatory network

To predict the interactions between differentially expressed mRNA and miRNA in ectopic endometrium samples, we utilized the miRNet database (https://www.mirnet.ca/). The mRNA-miRNA regulatory network was then visualized in Cytoscape software, providing an overview of the interactions between mRNAs and miRNAs, which can be considered potential targets.

### GO and KEGG pathway enrichment analyses of genes

The FRHGs were subjected to GO and KEGG pathway analysis using the ClusterProfiler package in R. The analysis considered three criteria: molecular function (MF), cellular component (CC), and biological process (BP). For statistical significance, an adjusted P value of 0.05 was used as per the Benjamini–Hochberg method, and the analysis was limited to *Homo sapiens*.

### Gene set enrichment analysis (GSEA)

To investigate biological signaling pathways, we conducted a GSEA analysis. We identified the KEGG pathway showing significant enrichment based on the net enrichment score (NES), gene ratio, and P value. We considered |NES| greater than one and FDR q less than 0.25 as indicators of significant enrichment.

### Validation of mitosis hub genes

In this study, we used 'pROC’ (version 1.18.0) and ‘ggplot2’ packages (version 3.3.6) of R software (version 4.2.1) to perform Receiver Operating Characteristic (ROC) curve analysis. The analysis helped us to determine the sensitivity and specificity of target genes. We quantified the results by calculating the area under the ROC curve (AUC). The genes with AUC >0.6 were considered diagnostic.

### Possible drugs for target genes

The DGIdb (Drug-Gene Interaction Database) is a web resource that helps organize genes of the druggable genome into known drug interactions and potentially druggable targets. We utilized module DEGs (Differentially Expressed Genes) of endometriosis and significantly evaluated DEGs in DGIdb to identify potentially druggable DEGs.

### Statistical analysis

Statistical analysis was performed using R version 4.2.1 and GraphPad Prism eight software. The data was presented as mean ± standard deviation, and an unpaired Student’s t-test was employed to compare groups. A P-value of less than 0.05 was considered statistically significant.

## Results

### Screening of mitosis-related hub genes in patients with endometriosis

**FIGURE 2:**
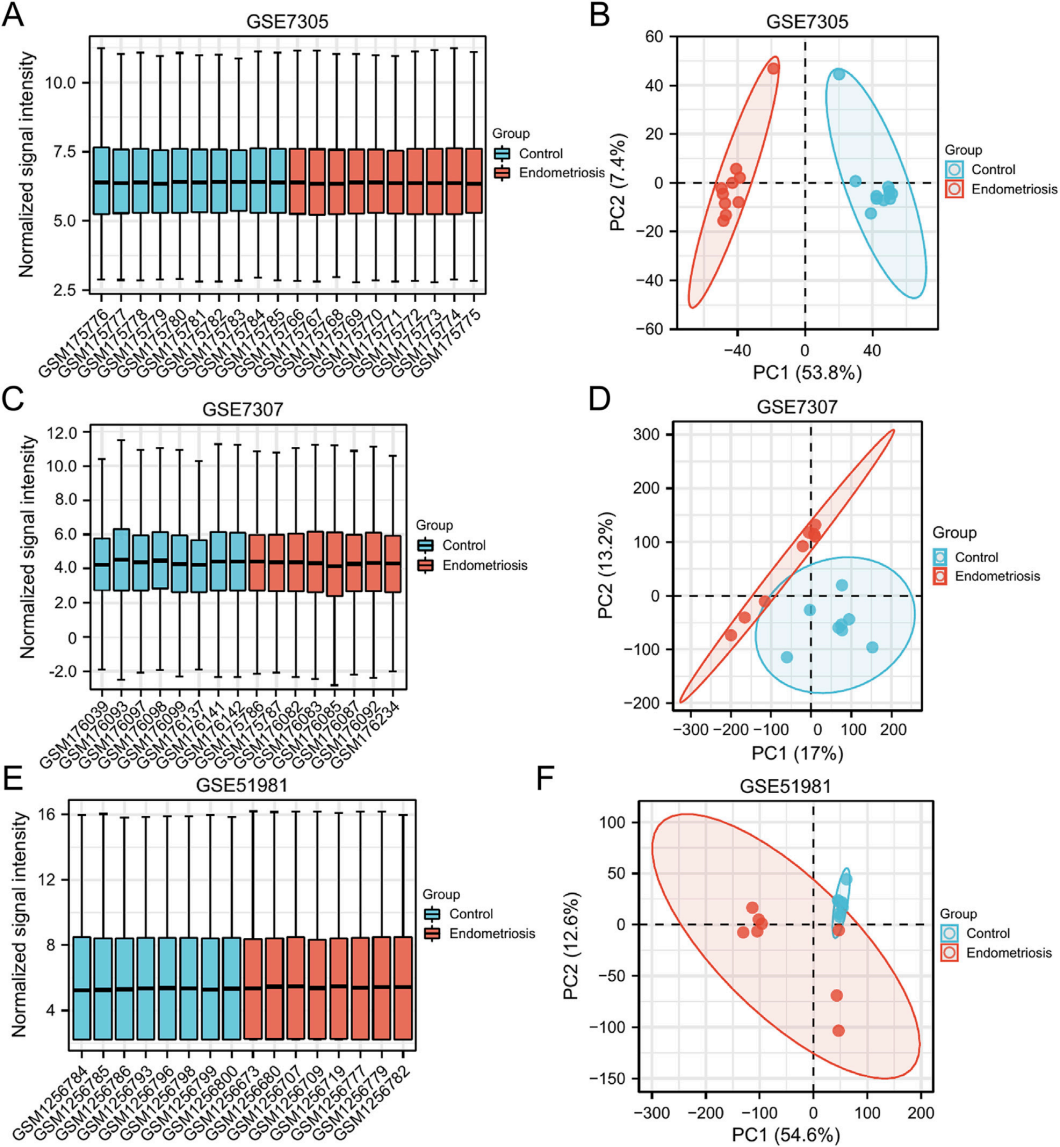
Normalized expression matrices **(A,C,E)** and PCA diagrams **(B,D,F)** of the GSE7305, GSE7307, and GSE51981 datasets. PCA, principal component analysis.

The expression matrix of GSE7305, GSE7307, and GSE51981 were normalized. The box plots showed that the distribution trend of the box graph is a straight line, and no library size effects were noticeable after normalization (Figures 2A,C,E). PCA of the three datasets was performed separately, and the PCA results that showed good repeatability of the data were visually compared (Figures 2B,D,F).

**FIGURE 3:**
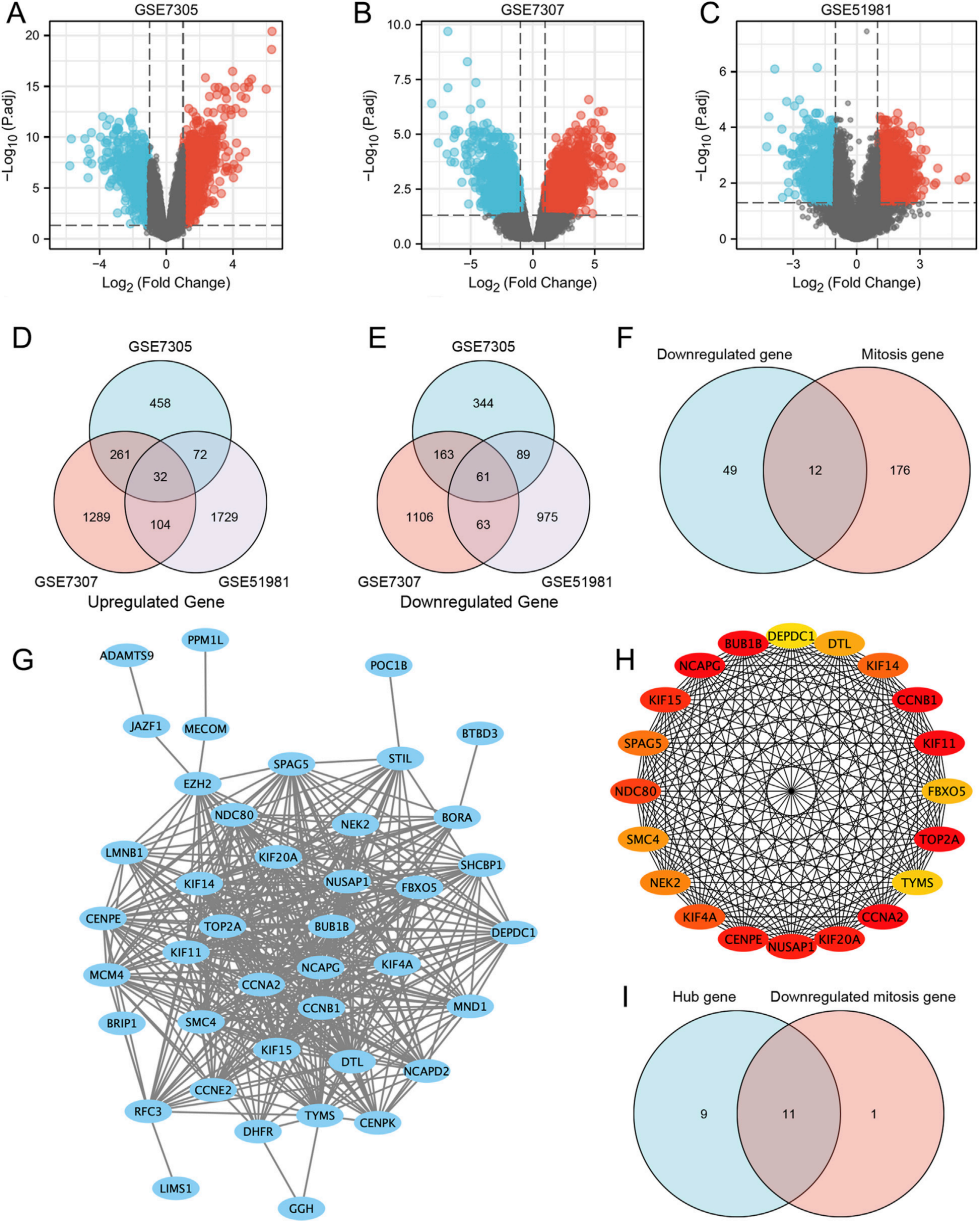
Differentially expressed genes and mitosis-related hub genes of the GSE7305, GSE7307, and GSE51981 datasets. **(A–C)** The volcano plots of GSE7305, GSE7307, and GSE51981; **(D)** Differential genes in the three datasets; **(E)** STRING; **(F)** Hub genes; **(G)** Mitosis-related genes; **(H)** Mitosis-related hub genes. **(I)** The genes that are both hub genes and differentially expressed genes in mitosis.

We identified 823 upregulated and 657 downregulated DEGs in the GSE7305 dataset, 1,686 upregulated and 1,393 downregulated DEGs in the GSE7307 dataset, and 1937 upregulated and 1,188 downregulated DEGs in the GSE51981 dataset. The volcano plots of DEGs in the above three datasets were generated in R, shown in Figures 3A–C. Venn plots showed that 93 genes were commonly differentially expressed among the three datasets (Figures 3D,E), of which 32 were upregulated, and 61 were downregulated (Supplementary Table S1).

Subsequently, we generated the PPI network (Figure 3G) by determining the interactions among the DEGs obtained above. Use Cytoscape (version 3.8.1) to import STRING analysis data and identify the top 20% of genes with the highest scores as hub genes. Finally, 20 hub genes were determined, which were: CCNA2, BUB1B, NCAPG, TOP2A, CCNB1, KIF11, CENPE, KIF20A, NUSAP1, KIF15, NDC80, KIF4A, KIF14, SPAG5, NEK2, SMC4, DTL, FBXO5, TYMS and DEPDC1 (Figure 3H).

In addition, we analyzed the expression of 12 mitosis-related downregulated genes in three datasets. Venn diagram analysis of the three data sets found that the genes BUB1B, KIF11, CCNB1, NDC80, NEK2, CCNA2, KIF4A, NCAPG, CENPE, KIF14, BORA and FBXO5 were downregulated genes associated with mitosis (Figure 3F). Interestingly, some of these genes overlap with the hub above genes, namely, BUB1B, KIF11, CCNB1, NDC80, NEK2, CCNA2, KIF4A, NCAPG, CENPE, KIF14, and FBXO5. They are both hub and differentially expressed genes in mitosis (Figure 3I).

### GSE25628 confirmed the expression and diagnostic value of the MRHGs

**FIGURE 4:**
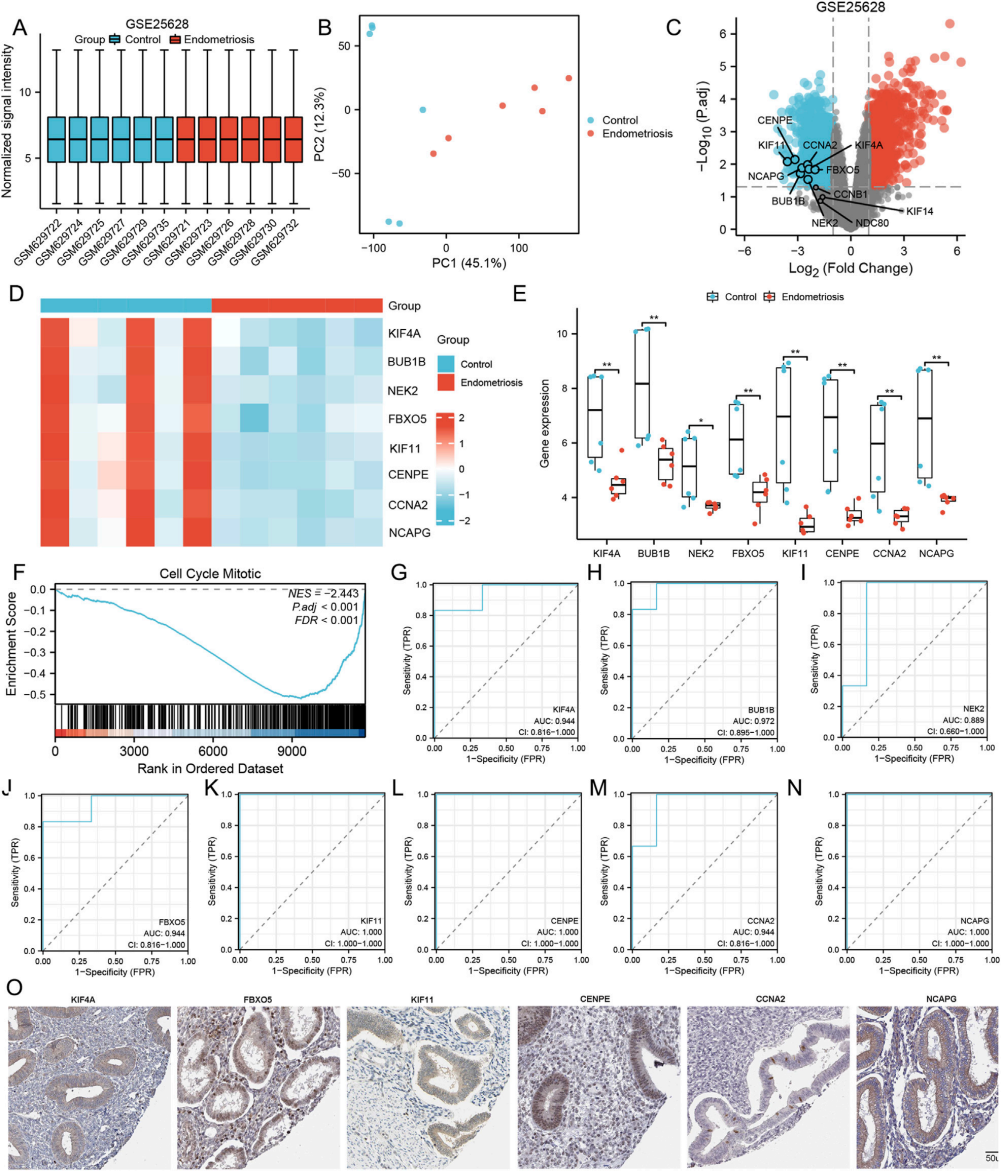
Comparison of the expression and diagnostic ROC curves of mitosis-related hub genes. **(A–D)** Normalized expression matrices, PCA diagrams, volcano plots, and heat map of GSE25628; **(E)** Comparison of the expression of mitosis-related hub genes in Control and Endometriosis samples; **(F)** Signaling pathways where the mitosis-related hub genes are predominant in Control and Endometriosis samples; **(G–N)** Diagnostic ROC curves of mitosis-related hub genes in Control and Endometriosis samples; **(O)** The protein endometrium localization of mitosis-related hub genes. **, P < 0.01. ROC is the receiver operating characteristic; TPR is the true positive rate; FPR is the false positive rate.

We used the GSE25628 dataset to detect the expression of selected target genes, and the results showed that the differential expression of eight MRHGs (KIF4A, BUB1B, NEK2, FBXO5, KIF11, CENPE, CCNA2, and NCAPG) between control and endometriosis patients was consistent with predictions (Figures 4A–E). Then, functional enrichment and identification of ectopic and normal endometrium by GSEA showed that most of the genes were located in the cell cycle mitotic pathway (NES = −2.443; P. adj <0.001; FDR <0.001) (Figure 4F). ROC curves using ectopic and normal endometrial data showed that these eight genes are of great value in diagnosing endometriosis. Figures 4G–N showed the AUC and 95% CI of eight MRHGs. The representative images were obtained from the Human Protein Atlas (HPA) and referenced to annotate the expression of these molecules in normal tissues (Figure 4O).

### GO/KEGG enrichment analyses of MRHGs and construction of the gene network

**FIGURE 5:**
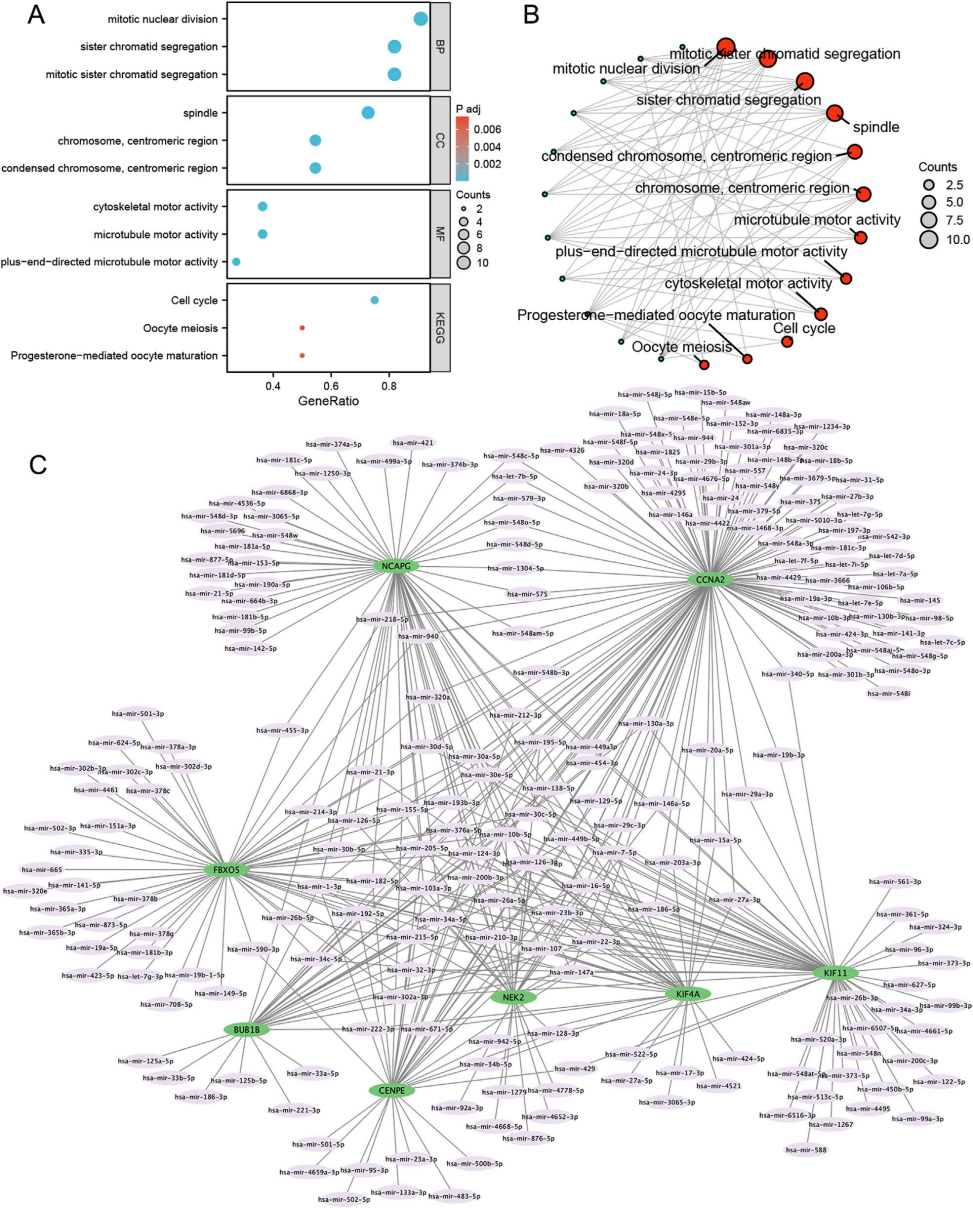
GO/KEGG and mRNA-miRNA regulatory network of mitosis-related hub genes. **(A,B)** GO categories and KEGG pathways; **(C)** mRNA-miRNA regulatory network. BP, biological process; MF, molecular function; GO, Gene Ontology; KEGG, Kyoto Encyclopedia of Genes and Genomes.

We performed GO and KEGG enrichment analyses on eight differentially expressed mitosis hub genes. The mitotic nuclear division and sister chromatid segregation were the most significant enrichment in GO categories. At the same time, the DEGs were mainly involved in the cell cycle, oocyte meiosis, and progesterone-mediated oocyte maturation in KEGG enrichment analysis (Figures 5A,B).

Using the miRNet tool, we obtained 235 target miRNAs of eight specifically expressed MRHGs and determined 420 mRNA-miRNA pairs. Then, according to the prediction results, we constructed a co-expressed network of mRNAs and miRNAs comprising 243 nodes and 420 edges (Figure 5C). BUB1B was regulated by 32 miRNAs, 115 miRNAs regulated CCNA2, 32 miRNAs held CENPE, 56 miRNAs controlled FBXO5, 61 miRNAs restrained KIF11, KIF4A was regulated by 34 miRNAs, 61 miRNAs regulated NCAPG, and NEK2 was hindered by 32 miRNAs (Supplementary Table S2). We identified 28 miRNAs by searching the PubMed database for literature on endometriosis and miRNAs mentioned above (Table 2).

**TABLE 2:** miRNAs associated with endometriosis reported in the pubmed database.

miRNA	Doi
hsa-let-7i-5p	10.1007/s43032-020-00148-z
hsa-mir-124-3p, hsa-mir-126-3p	10.1016/j.yexcr.2019.05.010
	10.1186/s12905-023-02250-1
	10.1007/s13258-021-01184-year
hsa-mir-125b-4p, hsa-mir-29a-3p	10.1093/humrep/dez116
hsa-mir-125b-5p	10.1016/j.fertnstert.2019.04.011
hsa-mir-15a-5p, hsa-mir-15b-5p, hsa-mir-16-5p, has-mir-195-5p, hsa-mir-320a, hsa-mir-320b, hsa-mir-92a-3p	0.1080/09537104.2022.2042233
hsa-mir-182-5p	10.1177/2,058,738,420,976,309
hsa-mir-203a-3p	10.1080/09,513,590.2022.2076830
hsa-mir-214-3p, hsa-mir-22-3p	10.1093/humrep/dead216
hsa-mir-218-5p	10.1186/s12958-022-00928-z
hsa-mir-24	10.1007/s00404-021-05963-6
hsa-mir-340-5p	10.1007/s00404-021-05963-6
hsa-mir-375	10.1007/s43032-022-00854-w
hsa-mir-378a-3p	10.1016/j.rbmo.2018.05.007
hsa-mir-421	10.1515/hmbci-2022-0039
hsa-mir-429	10.3892/mmr.2021.12055
hsa-mir-449a, hsa-mir-873-5p	10.5603/GP.a2022.0078
hsa-mir-483-5p	10.1186/1,477-7827-11-78
hsa-mir-502-5p	10.1080/07,391,102.2023.2291834

### Differential expression analysis of genes related to both endometrial decidualization and mitosis-related hub genes in GSE6364

**FIGURE 6:**
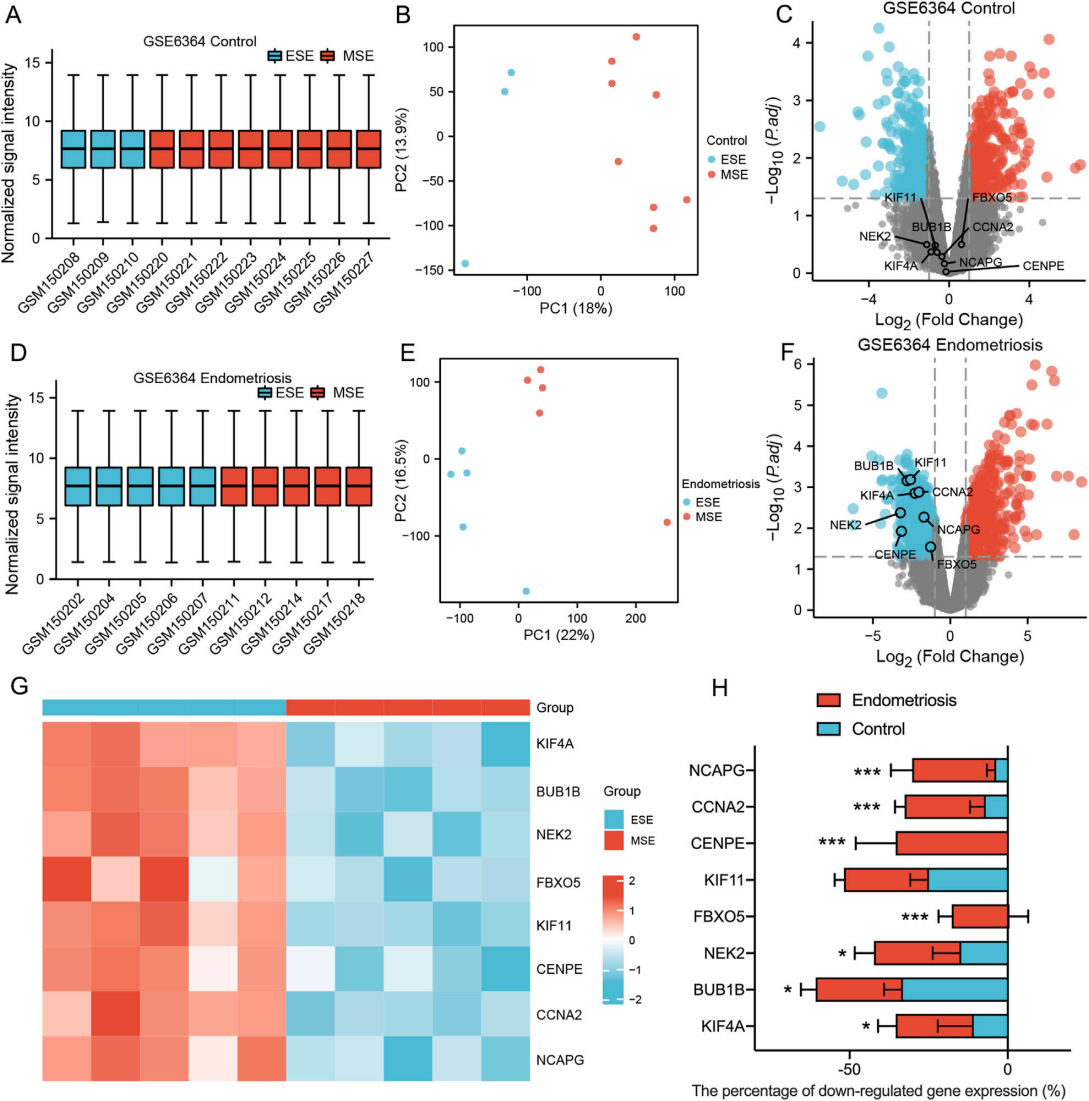
**(A–F)** Normalized expression matrices, principal component analysis, and volcano plots of 2 GSE6364 datasets (Control group ESE and MSE/Endometriosis group ESE and MSE); **(G)** The heat map of GSE6364 Endometriosis group; **(H)** The downregulation percentage of mitosis-related hub genes in Control and Endometriosis samples from ESE to MSE phase. ESE, Early Secretory; MSE, Mid-secretory. ***, P < 0.001, *, P < 0.05.

The transition of regularly cycling endometrium from a proliferative capacity to a differentiated 'decidual’ phenotype requires widespread changes in gene expression in preparation for implantation. This process appears delayed in women with severe endometriosis. The 8 MRHGs were tested with GSE6364, which contained early secretory (ESE) and mid-secretory (MSE) phases of endometrium from two groups (Control and endometriosis). The data showed no significant differences in 8 MRHGs of the endometrium in MSE and ESE in the control group (Figures 6A–C). However, they were significantly downregulated in endometriosis (Figures 6D–H).

### Differential expression analysis of genes related to both infertility and mitosis-related hub genes in GSE120103

**FIGURE 7:**
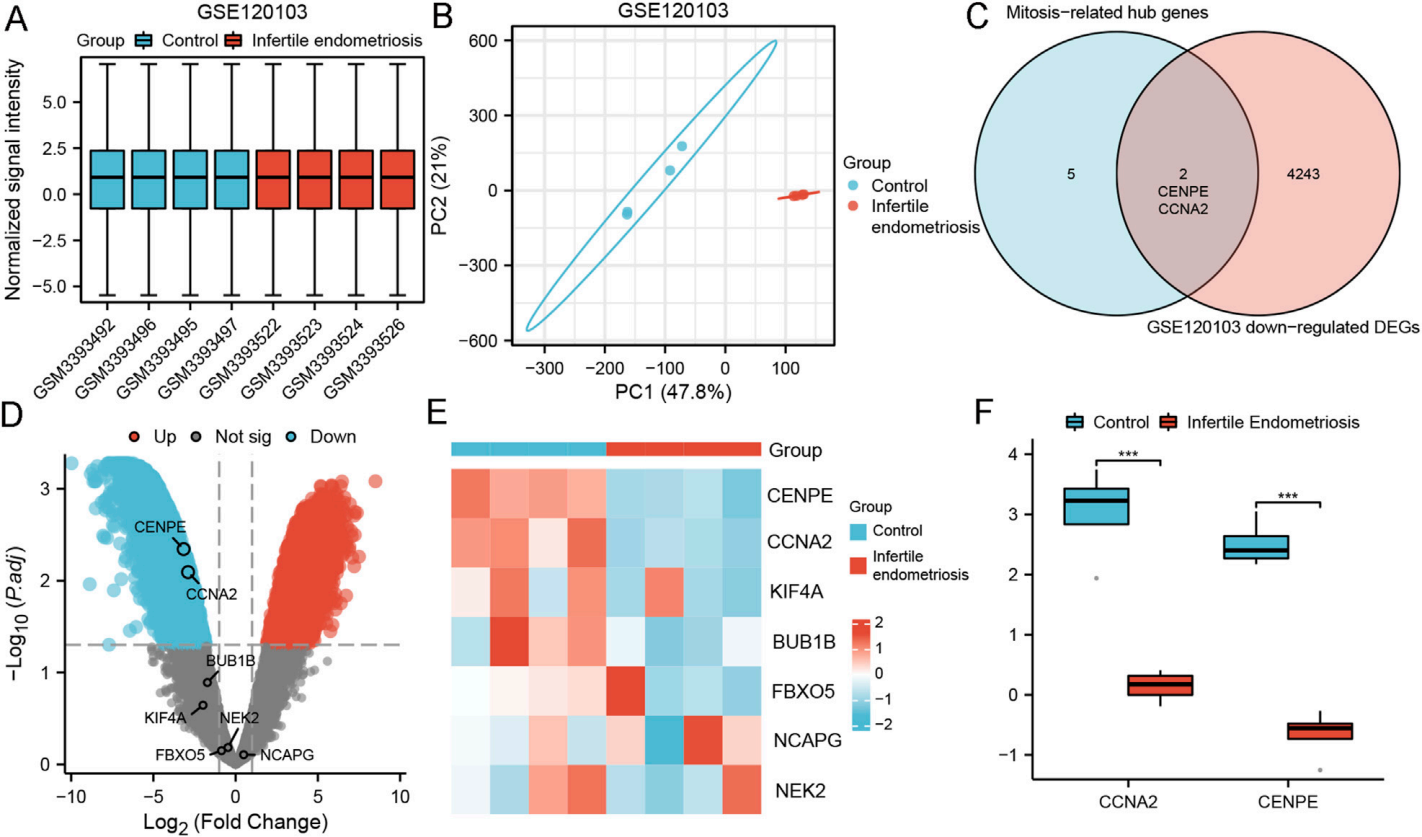
Differential expression analysis of genes related to infertility and mitosis-related hub genes in GSE120103. **(A,B)** Normalized expression matrices, principal component analysis; **(C)** Differential genes between mitosis-related hub genes and infertile endometriosis; **(D,E)** Volcano plots and heat map of mitosis-related hub genes in GSE120103; **(F)** Differences of mitosis-related hub genes in GSE120103; ***, P < 0.001.

Endometrial stromal cell decidualization was the key to embryo implantation. Impaired decidualization may lead to infertility. We further investigated the effect of endometriosis on fertility. We used the GSE120103 dataset containing ectopic endometrium of infertile endometriosis to detect the expression of screening target genes. The three datasets were normalized, and the distribution trends of box plots were straight lines (Figure 7A). PCA results showed good repeatability of the data (Figure 7B). Two MRHGs (CCNA2, CENPE) were downregulated in GSE120103, shown by Venn plots (Figure 7C). The volcano plots and heat maps of MRHGs were illustrated in Figures 7D,E. The statistical plots for individual genes are shown in Figure 7F. We finally found that CCNA2 and CENPE were related to infertile endometriosis.

### Differential expression analysis of genes related to infertility and mitosis targeted drug prediction

By regulating mitosis, the DGIdb database predicted potential target drugs associated with critical genes that may treat infertility endometriosis. Finally, eight target drugs were expected, and the data were shown in Table 3. One of the most noteworthy was cordycepin, which can inhibit the proliferation of human epithelial endometriosis cells, activate apoptosis, and promote the quality of aging of oocytes *in vitro* (Imesch et al., 2011; Li et al., 2023).

**TABLE 3:** Drug that may be effective against infertile endometriosis.

Drug	Regulatory approval	Indication	Interaction score
GSK-923295	Not Approved		58.9800539
Cordycepin	Not Approved		8.42572199
Genistein	Approved		0.32406623
Ethinyl estradiol	Approved	Contraceptive	0.99126141
Suramin	Not Approved		0.5617148
Seliciclib	Not Approved	Antineoplastic agent	1.404287
TNF-alpha	Not Approved		0.7021435
Tamoxifen	Approved	Hormonal, antineoplastic agent	0.27625318

In addition, it has been reported that cordycepin may be a potential drug for anti-aging and oxidative stress-induced male infertility (Kopalli et al., 2022).

## Discussion

Endometriosis is a chronic disease influenced by environmental and genetic factors, which has a high socioeconomic impact. The infertile endometriosis has apparent characteristics of reduced decidualization and impaired uterine receptivity. Endometrial receptivity depends on the precise and coordinated proliferation, differentiation, and apoptosis processes of endometrial stromal cells and glandular epithelial cells within the “implantation window” (6–10 days after ovulation) (Yang et al., 2022). This process is strictly regulated by Cyclins and cyclin-dependent kinases (CDKs). During the estrogen-dominated proliferative phase, the Cyclin D-CDK4/6 complex is required to drive cells from the G1 phase to the S phase (DNA replication), providing a basis for intimal thickening. If this stage is dysregulated (such as overexpression of Cyclin D), it may lead to excessive endometrial hyperplasia or endometriosis, and damage the receptivity of the endometrium. The secretory phase induced by progesterone relies on the Cyclin B-CDK1 complex to initiate mitosis, promote decidualization of intimal stromal cells, and differentiate them into glycogen-rich secretory cells, creating conditions for embryo adhesion. If inflammation or hormonal disorders lead to the inhibition of CDK1 activity, decidualization will be blocked and the receptivity of the endometrium will decline (Yusuf et al., 2023). Endometrial decidualization was the essential process to prepare the endometrium for pregnancy. Women with endometriosis are at increased risk of infertility by impacting the endometrial decidualization process (Giudice and Kao, 2004). Uterine stromal cell mitosis plays an essential role in this process and must undergo mitotic expansion before full decidualization (Wang et al., 2010; Muter et al., 2015). This study takes advantage of the similarity between endometriosis and infertility in mitosis to find a target for infertility treatment.

Based on the analysis of three data sets, GSE7305, GSE7307, and GSE51981, a PPI network was constructed in the GEO database, and DEGs screening were performed on the three data sets. We obtained 93 DGEs by taking the intersection of three endometriosis datasets. Then, we identified 11 potential mitosis-related downregulated hub genes, among which eight genes (KIF4A, BUB1B, NEK2, FBXO5, KIF11, CENPE, CCNA2, and NCAPG) showed good diagnostic properties of endometriosis and two genes (CCNA2, CENPE) were closely related to infertile endometriosis. The main enriched GO functions revealed that the cell cycle mitotic pathway may be the critical pathway in endometriosis. It has been reported that assessing mitosis rates provides additional diagnostic value for advanced endometriosis (Wetzk et al., 2022), which hints at the accuracy of the signaling pathway we predict.

There is significant evidence that eight hub genes related to mitosis play a crucial role in endometriosis or fertility.

It is reported that KIF4A is a member of the kinase protein superfamily and is involved in a series of cellular processes, such as chromosome aggregation and cytoplasmic division during mitosis. KIF4A may regulate the meiosis of mouse oocytes by affecting the precise separation of spindle tissue and chromosomes, and the loss of KIF4A may be related to aneuploidy of aging oocytes (Tang et al., 2018). KIF4A can bind to BUB1 to regulate the expression of BUB1. BUB1B is an oocyte-expressed growth factor essential for follicular development. Endometriosis harms granular cells, and MnBP concentrations are associated with endometriosis. MnBP may affect the function of granulocyte cells by altering the expressions of BUB1B, CDC20, and cyclin B1 genes (Jin and Ye, 2021; Chou et al., 2020; Racki and Richter, 2006). NEK2 is a protein that is crucial in regulating various cell cycle processes. Its overproduction can disrupt the natural development of male germ cells, leading to abnormalities (Rhee and Wolgemuth, 2002). FBXO5 has been identified as a genuine substrate of beta-Trcp1. It has been observed that beta-Trcp1 plays a vital role in regulating the proper sequence of meiotic and mitotic events. KIF11 is a type of kinesin composed of four identical subunits, and its protein expression is at its highest level during mitosis. If the recruitment of KIF11 on meiotic spindles is reduced, it could impair the proper functioning of these spindles (Guardavaccaro et al., 2003; Dong et al., 2023). CENPE is expressed during mitosis and plays critical roles in inaccurate chromosome alignment and as an emerging target for chemotherapy in clinical oncology (El-Arabey et al., 2018). The protein CCNA2 plays a crucial role in regulating the cell cycle progression, specifically in promoting cell proliferation. It is an indispensable component for the development of embryos (Chotiner et al., 2019). NCAPG is a critical component of condensin I, a protein complex that helps compact DNA during cell division. Its role in chromatin condensation during mitosis is well established. Recent studies have shown that NCAPG is also closely linked to fertility traits in Japanese Black cattle (Kawaguchi et al., 2020).

Co-expressed and miRNAs were chosen to construct mRNA-miRNA interaction networks to identify the critical miRNAs for the prognosis of endometriosis. As Figure 4 shows, BUB1B had 32 miRNAs, CCNA2 had 115 miRNAs, CENPE had 35 miRNAs, FBXO5 had 56 miRNAs, KIF11 had 61 miRNAs, KIF4A had 34 miRNAs, NCAPG had 61 miRNAs, NEK2 had 26 miRNAs. Endometriosis and fertility have been found to have links with multiple miRNAs. Numerous studies have reported on the association of miRNAs with these conditions. For example, Hsa-let-7i-5p, hsa-mir-125b-4p, and hsa-mir-29a-3p could be potential biomarkers and therapeutic targets for diagnosing and treating endometriosis (Gu et al., 2020; Vanhie et al., 2019). Moreover, miR-124-3p significantly regulates cell proliferation and invasion of ectopic endometrium through multiple pathways (Liu et al., 2019; Liu et al., 2023; Yuan et al., 2022). What is more, Hsa-mir-22-3p is not only an essential gene in granulosa cells of patients with biochemical primary ovarian insufficiency, which is a common condition leading to the pathological decline of ovarian function in women of reproductive age, resulting in infertility, but also a sensitive and specific indicator in distinguishing endometriosis from non-endometriosis (Liu et al., 2024; Nazri et al., 2023).

Although all eight screened MRHGs have been reported to mediate mitosis, it should be noted that there is a lack of evidence regarding their ability to regulate mitosis in endometriosis, especially in infertile endometriosis. Moreover, the sample size of infertile patients with endometriosis is limited, and more comprehensive investigations are necessary to analyze the underlying mechanisms in the future.

## Conclusion

Our study has identified eight potential mitosis hub genes (KIF4A, BUB1B, NEK2, FBXO5, KIF11, CENPE, CCNA2, and NCAPG) that exhibit excellent diagnostic properties for endometriosis. We have also constructed a network of associated mRNA-miRNA pathways. In addition, we have found that two of these hub genes (MRHGs) are related to infertile endometriosis and have shown high drug-targeting relevance for cordycepin. These findings provide new insights into the unique relationship between mitosis and endometriosis. However, they lack clinical certification. In future work, we will make full use of clinical samples for confirmation as much as possible.

Although the limited sample size is a challenge, our study provides a solid foundation for further research in the field of cross-cutting endometriosis and infertility. By addressing this limitation through collaborative efforts, validation studies, and integrating various data types, we can improve our understanding of the pathogenesis in which endometriosis damages female fertility and pave the way for improved diagnosis and targeted treatment of this condition.

## Data Availability

Publicly available datasets were analyzed in this study. This data can be found here: https://www.jianguoyun.com/p/DaEX4AAQuqSzDRiqv_UFIAA.
