# Supplementary material for: Identification of key genes associated with infertile endometriosis based on bioinformatic analysis
Source: Front Genet. 2025 Jul 23;16:1615268. doi: 10.3389/fgene.2025.1615268 (PMC12325009; doi:10.3389/fgene.2025.1615268)
Supplement: Supplementary file 1 [file Table2.docx]

**Table S1 Commonly differentially expressed among GSE7305, GSE7307 AND GSE51981**

| Gene Symbol | Up/Down | Gene Symbol | Up/Down | Gene Symbol | Up/Down |
| --- | --- | --- | --- | --- | --- |
| MYH11 | Upregulated | YPEL3 | Upregulated | CCNE2 | Downregulated |
| BST2 | Upregulated | TOP2A | Downregulated | DEPDC1 | Downregulated |
| KLF2 | Upregulated | CPM | Downregulated | ZNF367 | Downregulated |
| C10orf10 | Upregulated | UGT8 | Downregulated | PRSS12 | Downregulated |
| SERPINA3 | Upregulated | TYMS | Downregulated | GGH | Downregulated |
| TIMP1 | Upregulated | STX18 | Downregulated | MCM4 | Downregulated |
| CXCL2 | Upregulated | KIF20A | Downregulated | EZH2 | Downregulated |
| TNS1 | Upregulated | BUB1B | Downregulated | MMP16 | Downregulated |
| C11orf96 | Upregulated | ESRP1 | Downregulated | POC1B | Downregulated |
| S100A10 | Upregulated | DCDC2 | Downregulated | CENPE | Downregulated |
| FXYD6 | Upregulated | KIF11 | Downregulated | NLGN1 | Downregulated |
| AQP1 | Upregulated | FREM2 | Downregulated | TXN | Downregulated |
| RARRES2 | Upregulated | CCNB1 | Downregulated | KIF14 | Downregulated |
| NBL1 | Upregulated | NUSAP1 | Downregulated | MND1 | Downregulated |
| ADM | Upregulated | NDC80 | Downregulated | PTN | Downregulated |
| CLDN5 | Upregulated | CENPK | Downregulated | BRIP1 | Downregulated |
| CBX7 | Upregulated | NEK2 | Downregulated | SMC4 | Downregulated |
| FMOD | Upregulated | CCNA2 | Downregulated | JAZF1 | Downregulated |
| JUNB | Upregulated | ADAMTS9 | Downregulated | LIMS1 | Downregulated |
| IL4R | Upregulated | KIF15 | Downregulated | SPAG5 | Downregulated |
| RAPGEF3 | Upregulated | GRHL2 | Downregulated | RFC3 | Downregulated |
| PCED1B | Upregulated | PAPSS1 | Downregulated | LOC90246 | Downregulated |
| SERPINF1 | Upregulated | GREM2 | Downregulated | DHFR | Downregulated |
| RARRES3 | Upregulated | SHCBP1 | Downregulated | GPR137C | Downregulated |
| NFIX | Upregulated | KIF4A | Downregulated | BTBD3 | Downregulated |
| ENG | Upregulated | MECOM | Downregulated | STIL | Downregulated |
| APOE | Upregulated | DTL | Downregulated | BORA | Downregulated |
| QSOX1 | Upregulated | SPA17 | Downregulated | FBXO5 | Downregulated |
| ENO2 | Upregulated | GCNT2 | Downregulated | PPM1L | Downregulated |
| GATSL2 | Upregulated | LMNB1 | Downregulated | NCAPD2 | Downregulated |
| LRP1 | Upregulated | NCAPG | Downregulated | GPSM2 | Downregulated |

**Table S2 miRNA target gene**

| ID | Target | ID | Target | ID | Target | ID | Target |
| --- | --- | --- | --- | --- | --- | --- | --- |
| hsa-mir-22-3p | BUB1B | hsa-mir-34a-5p | CCNA2 | hsa-mir-34a-5p | FBXO5 | hsa-mir-4521 | KIF4A |
| hsa-mir-192-5p | BUB1B | hsa-mir-3679-5p | CCNA2 | hsa-mir-34c-5p | FBXO5 | hsa-mir-522-5p | KIF4A |
| hsa-mir-215-5p | BUB1B | hsa-mir-375 | CCNA2 | hsa-mir-365a-3p | FBXO5 | hsa-mir-942-5p | KIF4A |
| hsa-mir-193b-3p | BUB1B | hsa-mir-379-5p | CCNA2 | hsa-mir-365b-3p | FBXO5 | hsa-mir-103a-3p | KIF4A |
| hsa-mir-125a-5p | BUB1B | hsa-mir-424-3p | CCNA2 | hsa-mir-378g | FBXO5 | hsa-mir-103a-4p | KIF4A |
| hsa-mir-125b-5p | BUB1B | hsa-mir-4326 | CCNA2 | hsa-mir-423-5p | FBXO5 | hsa-mir-103a-5p | KIF4A |
| hsa-mir-186-3p | BUB1B | hsa-mir-4422 | CCNA2 | hsa-mir-4461 | FBXO5 | hsa-mir-103a-6p | KIF4A |
| hsa-mir-221-3p | BUB1B | hsa-mir-4429 | CCNA2 | hsa-mir-455-3p | FBXO5 | hsa-mir-103a-7p | KIF4A |
| hsa-mir-222-3p | BUB1B | hsa-mir-5010-3p | CCNA2 | hsa-mir-501-3p | FBXO5 | hsa-mir-103a-8p | KIF4A |
| hsa-mir-32-3p | BUB1B | hsa-mir-542-3p | CCNA2 | hsa-mir-502-3p | FBXO5 | hsa-mir-103a-9p | KIF4A |
| hsa-mir-33a-5p | BUB1B | hsa-mir-548a-3p | CCNA2 | hsa-mir-548b-3p | FBXO5 | hsa-mir-103a-10p | KIF4A |
| hsa-mir-33b-5p | BUB1B | hsa-mir-548am-5p | CCNA2 | hsa-mir-590-3p | FBXO5 | hsa-mir-103a-11p | KIF4A |
| hsa-mir-454-3p | BUB1B | hsa-mir-548b-3p | CCNA2 | hsa-mir-624-5p | FBXO5 | hsa-mir-103a-12p | KIF4A |
| hsa-mir-1-3p | BUB1B | hsa-mir-548c-5p | CCNA2 | hsa-mir-665 | FBXO5 | hsa-mir-103a-13p | KIF4A |
| hsa-mir-124-3p | BUB1B | hsa-mir-548d-5p | CCNA2 | hsa-mir-708-5p | FBXO5 | hsa-mir-103a-14p | KIF4A |
| hsa-mir-103a-3p | BUB1B | hsa-mir-548i | CCNA2 | hsa-mir-873-5p | FBXO5 | hsa-mir-103a-15p | KIF4A |
| hsa-mir-107 | BUB1B | hsa-mir-548j-5p | CCNA2 | hsa-mir-940 | FBXO5 | hsa-mir-103a-16p | KIF4A |
| hsa-mir-126-3p | BUB1B | hsa-mir-548o-3p | CCNA2 | hsa-mir-1-3p | FBXO5 | hsa-mir-103a-17p | KIF4A |
| hsa-mir-129-2-3p | BUB1B | hsa-mir-548o-5p | CCNA2 | hsa-mir-10b-5p | FBXO5 | hsa-mir-103a-18p | KIF4A |
| hsa-mir-130a-3p | BUB1B | hsa-mir-548y | CCNA2 | hsa-mir-124-3p | FBXO5 | hsa-mir-103a-19p | KIF4A |
| hsa-mir-138-5p | BUB1B | hsa-mir-557 | CCNA2 | hsa-mir-147a | FBXO5 | hsa-mir-103a-20p | KIF4A |
| hsa-mir-147a | BUB1B | hsa-mir-579-3p | CCNA2 | hsa-mir-205-5p | FBXO5 | hsa-mir-103a-21p | KIF4A |
| hsa-mir-16-5p | BUB1B | hsa-mir-7-5p | CCNA2 | hsa-mir-214-3p | FBXO5 | hsa-mir-103a-22p | KIF4A |
| hsa-mir-195-5p | BUB1B | hsa-mir-940 | CCNA2 | hsa-mir-376a-5p | FBXO5 | hsa-mir-103a-23p | KIF4A |
| hsa-mir-200b-3p | BUB1B | hsa-mir-944 | CCNA2 | hsa-mir-302a-3p | FBXO5 | hsa-mir-103a-24p | NCAPG |
| hsa-mir-205-5p | BUB1B | hsa-mir-23b-3p | CCNA2 | hsa-mir-302b-3p | FBXO5 | hsa-mir-103a-25p | NCAPG |
| hsa-mir-214-3p | BUB1B | hsa-mir-1-3p | CCNA2 | hsa-mir-302c-3p | FBXO5 | hsa-mir-103a-26p | NCAPG |
| hsa-mir-26a-5p | BUB1B | hsa-mir-10b-5p | CCNA2 | hsa-mir-302d-3p | FBXO5 | hsa-mir-103a-27p | NCAPG |
| hsa-mir-34a-5p | BUB1B | hsa-mir-124-3p | CCNA2 | hsa-mir-320e | FBXO5 | hsa-mir-103a-28p | NCAPG |
| hsa-mir-376a-5p | BUB1B | hsa-mir-126-3p | CCNA2 | hsa-mir-378a-3p | FBXO5 | hsa-mir-103a-29p | NCAPG |
| hsa-mir-449b-5p | BUB1B | hsa-mir-129-2-3p | CCNA2 | hsa-mir-378b | FBXO5 | hsa-mir-103a-30p | NCAPG |
| hsa-mir-671-5p | BUB1B | hsa-mir-147a | CCNA2 | hsa-mir-378c | FBXO5 | hsa-mir-103a-31p | NCAPG |
| hsa-let-7b-5p | CCNA2 | hsa-mir-155-5p | CCNA2 | hsa-mir-30a-5p | KIF11 | hsa-mir-103a-32p | NCAPG |
| hsa-mir-19a-3p | CCNA2 | hsa-mir-203a-3p | CCNA2 | hsa-mir-30c-5p | KIF11 | hsa-mir-103a-33p | NCAPG |
| hsa-mir-19b-3p | CCNA2 | hsa-mir-205-5p | CCNA2 | hsa-mir-30d-5p | KIF11 | hsa-mir-103a-34p | NCAPG |
| hsa-mir-22-3p | CCNA2 | hsa-mir-31-5p | CCNA2 | hsa-mir-34a-5p | KIF11 | hsa-mir-103a-35p | NCAPG |
| hsa-mir-24-3p | CCNA2 | hsa-mir-376a-5p | CCNA2 | hsa-mir-30b-5p | KIF11 | hsa-mir-103a-36p | NCAPG |
| hsa-mir-29a-3p | CCNA2 | hsa-mir-449a | CCNA2 | hsa-mir-186-5p | KIF11 | hsa-mir-103a-37p | NCAPG |
| hsa-mir-98-5p | CCNA2 | hsa-mir-449b-5p | CCNA2 | hsa-mir-30e-5p | KIF11 | hsa-mir-103a-38p | NCAPG |
| hsa-mir-29b-3p | CCNA2 | hsa-mir-18a-5p | CCNA2 | hsa-mir-193b-3p | KIF11 | hsa-mir-103a-39p | NCAPG |
| hsa-mir-197-3p | CCNA2 | hsa-mir-18b-5p | CCNA2 | hsa-mir-96-3p | KIF11 | hsa-mir-103a-40p | NCAPG |
| hsa-mir-148a-3p | CCNA2 | hsa-mir-24 | CCNA2 | hsa-mir-340-5p | KIF11 | hsa-mir-103a-41p | NCAPG |
| hsa-mir-212-3p | CCNA2 | hsa-mir-145 | CCNA2 | hsa-mir-4495 | KIF11 | hsa-mir-103a-42p | NCAPG |
| hsa-mir-27b-3p | CCNA2 | hsa-mir-146a | CCNA2 | hsa-mir-6507-5p | KIF11 | hsa-mir-103a-43p | NCAPG |
| hsa-mir-130a-3p | CCNA2 | hsa-mir-192-5p | CENPE | hsa-mir-6516-3p | KIF11 | hsa-mir-103a-44p | NCAPG |
| hsa-mir-132-3p | CCNA2 | hsa-mir-215-5p | CENPE | hsa-mir-122-5p | KIF11 | hsa-mir-103a-45p | NCAPG |
| hsa-mir-152-3p | CCNA2 | hsa-mir-129-5p | CENPE | hsa-mir-1267 | KIF11 | hsa-mir-103a-46p | NCAPG |
| hsa-mir-146a-5p | CCNA2 | hsa-mir-23a-3p | CENPE | hsa-mir-200b-3p | KIF11 | hsa-mir-103a-47p | NCAPG |
| hsa-mir-29c-3p | CCNA2 | hsa-mir-23b-3p | CENPE | hsa-mir-200c-3p | KIF11 | hsa-mir-103a-48p | NCAPG |
| hsa-mir-301a-3p | CCNA2 | hsa-mir-34b-5p | CENPE | hsa-mir-22-3p | KIF11 | hsa-mir-103a-49p | NCAPG |
| hsa-mir-130b-3p | CCNA2 | hsa-mir-4659a-3p | CENPE | hsa-mir-26b-3p | KIF11 | hsa-mir-103a-50p | NCAPG |
| hsa-mir-148b-3p | CCNA2 | hsa-mir-500b-5p | CENPE | hsa-mir-27a-3p | KIF11 | hsa-mir-103a-51p | NCAPG |
| hsa-mir-193b-3p | CCNA2 | hsa-mir-501-5p | CENPE | hsa-mir-302a-3p | KIF11 | hsa-mir-103a-52p | NCAPG |
| hsa-mir-575 | CCNA2 | hsa-mir-502-5p | CENPE | hsa-mir-32-3p | KIF11 | hsa-mir-103a-53p | NCAPG |
| hsa-mir-454-3p | CCNA2 | hsa-mir-590-3p | CENPE | hsa-mir-324-3p | KIF11 | hsa-mir-103a-54p | NCAPG |
| hsa-mir-10b-3p | CCNA2 | hsa-mir-942-5p | CENPE | hsa-mir-34a-3p | KIF11 | hsa-mir-103a-55p | NCAPG |
| hsa-mir-301b-3p | CCNA2 | hsa-mir-1-3p | CENPE | hsa-mir-361-5p | KIF11 | hsa-mir-103a-56p | NCAPG |
| hsa-mir-1825 | CCNA2 | hsa-mir-103a-3p | CENPE | hsa-mir-373-3p | KIF11 | hsa-mir-103a-57p | NCAPG |
| hsa-mir-4295 | CCNA2 | hsa-mir-107 | CENPE | hsa-mir-373-5p | KIF11 | hsa-mir-103a-58p | NCAPG |
| hsa-mir-3666 | CCNA2 | hsa-mir-10b-5p | CENPE | hsa-mir-429 | KIF11 | hsa-mir-103a-59p | NCAPG |
| hsa-mir-4676-5p | CCNA2 | hsa-mir-124-3p | CENPE | hsa-mir-450b-5p | KIF11 | hsa-mir-103a-60p | NCAPG |
| hsa-mir-548aw | CCNA2 | hsa-mir-126-3p | CENPE | hsa-mir-4661-5p | KIF11 | hsa-mir-103a-61p | NCAPG |
| hsa-mir-548g-5p | CCNA2 | hsa-mir-128-3p | CENPE | hsa-mir-513c-5p | KIF11 | hsa-mir-103a-62p | NCAPG |
| hsa-mir-548x-5p | CCNA2 | hsa-mir-129-2-3p | CENPE | hsa-mir-520a-3p | KIF11 | hsa-mir-103a-63p | NCAPG |
| hsa-mir-548aj-5p | CCNA2 | hsa-mir-147a | CENPE | hsa-mir-522-5p | KIF11 | hsa-mir-103a-64p | NCAPG |
| hsa-mir-1468-3p | CCNA2 | hsa-mir-155-5p | CENPE | hsa-mir-548at-5p | KIF11 | hsa-mir-103a-65p | NCAPG |
| hsa-mir-548e-5p | CCNA2 | hsa-mir-16-5p | CENPE | hsa-mir-548n | KIF11 | hsa-mir-103a-66p | NCAPG |
| hsa-mir-548f-5p | CCNA2 | hsa-mir-182-5p | CENPE | hsa-mir-561-3p | KIF11 | hsa-mir-103a-67p | NCAPG |
| hsa-mir-6835-3p | CCNA2 | hsa-mir-195-5p | CENPE | hsa-mir-588 | KIF11 | hsa-mir-103a-68p | NCAPG |
| hsa-let-7a-5p | CCNA2 | hsa-mir-205-5p | CENPE | hsa-mir-627-5p | KIF11 | hsa-mir-103a-69p | NCAPG |
| hsa-let-7c-5p | CCNA2 | hsa-mir-210-3p | CENPE | hsa-mir-99a-3p | KIF11 | hsa-mir-103a-70p | NCAPG |
| hsa-let-7d-5p | CCNA2 | hsa-mir-212-3p | CENPE | hsa-mir-99b-3p | KIF11 | hsa-mir-103a-71p | NCAPG |
| hsa-let-7e-5p | CCNA2 | hsa-mir-29c-3p | CENPE | hsa-mir-1-3p | KIF11 | hsa-mir-103a-72p | NCAPG |
| hsa-let-7f-5p | CCNA2 | hsa-mir-34a-5p | CENPE | hsa-mir-103a-3p | KIF11 | hsa-mir-103a-73p | NCAPG |
| hsa-let-7g-5p | CCNA2 | hsa-mir-429 | CENPE | hsa-mir-107 | KIF11 | hsa-mir-103a-74p | NCAPG |
| hsa-let-7i-5p | CCNA2 | hsa-mir-449b-5p | CENPE | hsa-mir-10b-5p | KIF11 | hsa-mir-103a-75p | NCAPG |
| hsa-mir-103a-3p | CCNA2 | hsa-mir-483-5p | CENPE | hsa-mir-124-3p | KIF11 | hsa-mir-103a-76p | NCAPG |
| hsa-mir-106b-5p | CCNA2 | hsa-mir-95-3p | CENPE | hsa-mir-126-3p | KIF11 | hsa-mir-103a-77p | NCAPG |
| hsa-mir-107 | CCNA2 | hsa-mir-133a-3p | CENPE | hsa-mir-128-3p | KIF11 | hsa-mir-103a-78p | NCAPG |
| hsa-mir-1234-3p | CCNA2 | hsa-mir-26b-5p | FBXO5 | hsa-mir-129-2-3p | KIF11 | hsa-mir-103a-79p | NCAPG |
| hsa-mir-129-5p | CCNA2 | hsa-mir-192-5p | FBXO5 | hsa-mir-130a-3p | KIF11 | hsa-mir-103a-80p | NCAPG |
| hsa-mir-1304-5p | CCNA2 | hsa-mir-215-5p | FBXO5 | hsa-mir-147a | KIF11 | hsa-mir-103a-81p | NCAPG |
| hsa-mir-138-5p | CCNA2 | hsa-mir-151a-3p | FBXO5 | hsa-mir-16-5p | KIF11 | hsa-mir-103a-82p | NCAPG |
| hsa-mir-141-3p | CCNA2 | hsa-mir-193b-3p | FBXO5 | hsa-mir-182-5p | KIF11 | hsa-mir-103a-83p | NCAPG |
| hsa-mir-15a-5p | CCNA2 | hsa-let-7g-3p | FBXO5 | hsa-mir-203a-3p | KIF11 | hsa-mir-103a-84p | NCAPG |
| hsa-mir-15b-5p | CCNA2 | hsa-mir-103a-3p | FBXO5 | hsa-mir-205-5p | KIF11 | hsa-mir-103a-85p | NEK2 |
| hsa-mir-16-5p | CCNA2 | hsa-mir-107 | FBXO5 | hsa-mir-210-3p | KIF11 | hsa-mir-103a-86p | NEK2 |
| hsa-mir-181c-3p | CCNA2 | hsa-mir-141-5p | FBXO5 | hsa-mir-26a-5p | KIF11 | hsa-mir-103a-87p | NEK2 |
| hsa-mir-186-5p | CCNA2 | hsa-mir-149-5p | FBXO5 | hsa-mir-29a-3p | KIF11 | hsa-mir-103a-88p | NEK2 |
| hsa-mir-195-5p | CCNA2 | hsa-mir-155-5p | FBXO5 | hsa-mir-449a | KIF11 | hsa-mir-103a-89p | NEK2 |
| hsa-mir-200a-3p | CCNA2 | hsa-mir-181b-3p | FBXO5 | hsa-mir-449b-5p | KIF11 | hsa-mir-103a-90p | NEK2 |
| hsa-mir-20a-5p | CCNA2 | hsa-mir-182-5p | FBXO5 | hsa-mir-19b-3p | KIF11 | hsa-mir-103a-91p | NEK2 |
| hsa-mir-218-5p | CCNA2 | hsa-mir-19a-5p | FBXO5 | hsa-mir-132-3p | KIF11 | hsa-mir-103a-92p | NEK2 |
| hsa-mir-27a-3p | CCNA2 | hsa-mir-19b-1-5p | FBXO5 | hsa-mir-26b-5p | KIF4A | hsa-mir-103a-93p | NEK2 |
| hsa-mir-30a-5p | CCNA2 | hsa-mir-218-5p | FBXO5 | hsa-mir-34a-5p | KIF4A | hsa-mir-103a-94p | NEK2 |
| hsa-mir-30c-5p | CCNA2 | hsa-mir-30a-5p | FBXO5 | hsa-mir-222-3p | KIF4A | hsa-mir-103a-95p | NEK2 |
| hsa-mir-30d-5p | CCNA2 | hsa-mir-30b-5p | FBXO5 | hsa-mir-1-3p | KIF4A | hsa-mir-103a-96p | NEK2 |
| hsa-mir-30e-5p | CCNA2 | hsa-mir-30c-5p | FBXO5 | hsa-mir-138-5p | KIF4A | hsa-mir-103a-97p | NEK2 |
| hsa-mir-320a | CCNA2 | hsa-mir-30d-5p | FBXO5 | hsa-mir-15a-5p | KIF4A | hsa-mir-103a-98p | NEK2 |
| hsa-mir-320b | CCNA2 | hsa-mir-30e-5p | FBXO5 | hsa-mir-17-3p | KIF4A | hsa-mir-103a-99p | NEK2 |
| hsa-mir-320c | CCNA2 | hsa-mir-320a | FBXO5 | hsa-mir-27a-3p | KIF4A | hsa-mir-103a-100p | NEK2 |
| hsa-mir-320d | CCNA2 | hsa-mir-32-3p | FBXO5 | hsa-mir-3065-3p | KIF4A |  |  |
| hsa-mir-340-5p | CCNA2 | hsa-mir-335-3p | FBXO5 | hsa-mir-424-5p | KIF4A |  |  |
